# Health Literacy of Rural Population of Kazakhstan

**DOI:** 10.18502/ijph.v49i7.3580

**Published:** 2020-07

**Authors:** Syrym S. SHAYAKHMETOV, Karlygash K. TOGUZBAYEVA, Aigul A. ISMAILOVA, Ramin TABIBI, Zhypar K. DERBISHALIEVA, Kenesh O. DZHUSUPOV

**Affiliations:** 1. Department of Public Health, Semey State Medical University, Semey, Republic of Kazakhstan; 2. Department of Nutrition and Preventive Medicine, JSC National Medical University, Almaty, Republic of Kazakhstan; 3. Department of Ecology, Seyfullin Agrotechnical University, Nur-Sultan, Republic of Kazakhstan; 4. School of Medicine, Abadan University of Medical Sciences, Abadan, Iran; 5. Department of Public Health, International School of Medicine, Bishkek, Kyrgyzstan

**Keywords:** Health literacy, Rural population, Kazakhstan

## Abstract

**Background:**

To date, there is no data available of health literacy of the population in Kazakhstan. This study was aimed to assess the health literacy of the rural population for the development of the targeted health education programs.

**Methods:**

The adapted HLS-EU-Q47 survey was carried out among 1650 respondents aged 18–76 from rural settlements in Almaty region of Kazakhstan in 2013. The health literacy competences to assess, understand, appraise and apply health information on healthcare, disease prevention and health promotion were measured. The associations between the health literacy competencies and demographic and socio-economic characteristics were shown through a multiple linear regression analysis.

**Results:**

The overall health literacy rate of the rural population of Almaty region was problematic and inadequate. With regards to their age, sex, social and economic characteristics, the health literacy competencies differ according to health literacy domain. Respondents with low education level or perceived social status had respectively low health literacy scores, especially in appraising and applying information of disease prevention.

**Conclusion:**

Low educated people and with lower income have lower health literacy in comparison to respondents with higher education level and higher income. Respondents with higher health literacy have higher rate of self-assessed health.

## Introduction

Health information is essential for a population to understand different aspects of own health and promote it. Currently, there are enormous amount of health information and information technologies accessed freely by the population. However, the use of this information and technologies depends heavily on the health literacy of the population (1–4). Health literacy is considered as a key factor in regards to personal “assets” and clinical “risk” (5), and health inequalities (6–8).

Knowledge of health literacy level of a population is important for health promotion and preventive health programs. Health literacy includes knowledge, motivation and activation, and it is complex to measure and influence. The elaboration of appropriate health literacy policy is based on the evidence on the extent, patterns and impact of low health literacy (3).

Low social and cognitive skills lead to low access, incorrect understanding and judging, and difficult application of health information and, finally, effect the person’s health behavior and health status. Low literacy is associated with different adverse health outcomes, including increased mortality, hospitalization, and in some cases poorer control of chronic health conditions (9–13).

During the last years, significant interest was observed in the defining health literacy (9–17). A range of tools have been offered and used to measure health literacy (19–23).

The concept of health literacy is new for post-soviet countries. In Kazakhstan, a small number of studies for assessment of people’s knowledge of health and risk-factors was carried out (24–26). Most of these studies used own concepts of health knowledge but not standardized measuring scale. Until present, there was no research to measure health literacy.

In the frame of the rural health project of the Kazakh National Medical University, during 2012–2013 (27), we attempted to study health literacy of the rural population using adapted version of the well-known HLS-EU-Q47.

This study was aimed to assess the health literacy of the rural population for the development of targeted health education programs.

## Methods

### Study settings

This cross-sectional study was conducted in the rural areas of Karasai rayon of Almaty region. The rural population of Karasai rayon was 149590 in 2013.

### Sampling and data collection

The size of a stratified random sample was calculated using the formula (28):

$$\text{s}={\text{X}}^{2}\text{NP}(1-\text{P})÷{\text{d}}^{2}(\text{N}-1)+{\text{X}}^{2}\text{P}(1-\text{P})$$

where s - required sample size; X2 – Chi-square for the specified confidence level at 1 degree of freedom; N – population size; P – population proportion.

The sample consisted of 1650 individuals of both sexes, aged 18 to 76 years. After exclusion of those with missing data, 1165 respondents were remained in the sample with full-completed forms for the further analysis. The survey was self-administered. The questionnaire was validated and distributed either in Russian or Kazakh languages.

### Questionnaire

The questionnaire was consisted of five parts. The first part included demographic and socioeconomic information including age, sex, educational level, marital status, ethnicity, household income per capita/month in tenge (KZT, Kazakh currency, 1 Euro=195.35 KZT as for Jun 2013) and perceived social status.

Educational level was defined as respondents who finished primary school only (1–4 years), secondary school (8 years of schooling), high school (11–12 years of schooling), and university degree (at least bachelor degree).

By the monthly household income, the respondents were grouped into 5 levels: less than 15,000, 15,000–20,000, 20,000–30,000, 30,000–40,000 and 40000 or more KZT/month per capita.

Self-reported perceived social status is used as one of the most accurate indicators of the social position (23, 29). The participants evaluated their perceived social status as low, medium and high.

The second part of the survey consisted of questions about life style factors that were physical activity, smoking, alcohol and drug use, and dietary habits.

The third part of the questionnaire included the 47-item health literacy scale form HLS-EU-Q47 to measure the rural population’s health literacy (HL) (16, 22). This form was developed by the HLS-EU consortium and based on the conceptual model including four health literacy competences and domains (16) of processing information: accessing, understanding, appraising, and applying information to make decisions in three areas of health: health care, disease prevention, and health promotion. In 50 point scale, those who answered “easy” or “very easy” for up to half of the questionnaire [0–25] would have inadequate health literacy; those who could answer “very easy” or “easy” up to 66% of the questionnaire [26–33] would have a problematic level; those who answered “easy” or “very easy” for up to 80% of the questionnaire [33–42] would be at the sufficient level; and those who answered “easy” or “very easy” for more than 80 percent of the questionnaire [42–50] would have excellent health literacy.

The fourth part of the questionnaire was devoted to the knowledge of health information on example of prevention of HIV/AIDS (human immunodeficiency virus/acquired immunodeficiency syndrome) and sexually transmitted diseases.

In the short fifth part of the questionnaire, respondents gave self-assessment of their health conditions.

### Ethics

The study was approved by the local Ethical Committee at the National Medical University named after S.D. Asfendiyarov. Participation in the study was voluntary and all the respondents signed an informed consent form.

### Statistical analysis

The reliability and internal consistency of the questionnaire were assessed using Cronbach’s alpha test, where a value of ≥0.7 was considered as satisfactory (30, 31). The internal consistency of the questionnaire items (to access, understand, appraise and apply information; to know prevention of HIV) was satisfactory: α=0.79, 0.81, 0.77, 0.84 and 0.79 respectively.

To compare the percentages of affirmative answers between different groups the chi-square test was used.

To explore the associations between the health literacy competencies and demographic and socio-economic characteristics (age, sex, ethnicity, education level, income level, perceived social status, smoking and alcohol abuse habits) a multiple linear regression analysis was used. Separately, to establish the correlation between ordinal variables and health literacy rates, the Kendall’s rank correlation coefficient τ (tau) was calculated with *P*-values (31).

These research data were processed using a package of standard statistical program SPSS 16.0 (Chicago, Illinois, USA).

## Results

The characteristics of the respondents are presented in the Table 1. Description of health literacy of the studied population is given in the Table 2. The presented scores show that the rural population perceives accessing, understanding, appraising and applying health information between “difficult” and “very easy”. The lowest literacy competence score (2.1) was for applying information in disease prevention. The highest score (3.4) was for understanding information in disease prevention. At the same time, the lowest, inadequate health literacy index (22.8) was for appraising health information, the highest but problematic–for the understanding health information (29.7). Among three health literacy domains the lowest, inadequate HL index was in the domain of health promotion (24.6) and the highest, problematic - in disease prevention domain, 27.9; for health care domain the HL index was also problematic – 26.5.

**Table 1:** Characteristics of the respondents

***Characteristics***	***Men (n=568)***		***Women (n=597)***		***Total (n=1165)***	
	***n***	***%***	***n***	***%***	***n***	***%***
Age group(yr):						
18–24	108	19.01	111	18.59	219	18.80
25–34	101	17.78	105	17.59	206	17.68
35–44	98	17.25	101	16.92	199	17.08
45–54	77	13.56	83	13.90	160	13.73
55–64	74	13.03	76	12.73	150	12.88
65–74	68	11.97	75	12.56	143	12.27
75 +	42	7.39	46	7.71	88	7.55
Marital status:						
Married	320	56.34	327	54.77	647	55.54
Single	187	32.92	199	33.33	386	33.13
Divorced	42	7.39	54	9.05	96	8.24
Widowed	19	3.35	17	2.85	36	3.09
Education level:						
Primary school	0	0.00	0	0.00	0	0.00
Secondary school	40	7.04	39	6.53	79	6.78
High school	483	85.04	507	84.92	990	84.98
University	45	7.92	51	8.54	96	8.24
Ethnic groups:						
Kazakhs	132	23.24	131	21.94	263	22.58
Russians	107	18.84	115	19.26	222	19.06
Uighurs	104	18.31	109	18.26	213	18.28
Turkish	101	17.78	105	17.59	206	17.68
Others	124	21.83	137	22.95	261	22.40
Income per capita (tenge/month)						
<15,000	39	6.87	68	11.39	107	9.18
15000 – 20000	97	17.08	104	17.42	201	17.25
20000 – 30000	195	34.33	199	33.33	394	33.82
30000 –40000	186	32.75	181	30.32	367	31.50
≥ 40000	51	8.98	45	7.54	96	8.24

**Table 2:** Descriptive statistics of health literacy of rural population in Kazakhstan (n=1165)

***Health literacy competence***	***Access information***		***Understand information***		***Appraise information***		***Apply information***		***HL index***	
	***Mean***	***SD***	***Mean***	***SD***	***Mean***	***SD***	***Mean***	***SD***	***Mean***	***SD***
Health care	2.3	0.61	2.6	0.52	2.4	0.57	2.7	0.22	26.5	5.8
Disease prevention	2.7	0.48	3.4	0.48	2.2	0.54	2.1	0.41	27.9	6.1
Health promotion	2.5	0.63	2.5	0.57	2.6	0.56	2.6	0.61	24.6	6.1
HL index	24.8	6.11	29.7	5.44	22.8	5.98	28.6	5.25	26.3	6.1

Inadequate HL index was found in 35% of all respondents with variation from 20.6 to 24.9. More than half of the respondents (60.6%) showed problematic health literacy (25.2–32.8) and only 4.5% of respondents had sufficient health literacy (33.4–40.9). No respondent showed excellent HL index. The general health literacy of surveyed rural population was problematic and made 26.3.

The associations between demographic, social and economic characteristics and health literacy of respondents are presented in Table 3. The results of multiple regression analysis show that there are some associations of demographic, social and economic determinants and health literacy. The strong association was found between education (secondary school) and health literacy, and there was some consistent association between smoking and alcohol abuse (in the domain of appraising health information).

No other variable had strong influence on health literacy (Table 3). Generally, respondents with lower education level, as well as with lower perceived social status had more difficulties with health literacy.

**Table 3:** Demographic, social and economic determination of health literacy of rural population in Kazakhstan (n=1165)

	***Access information***		***Understand information***		***Appraise information***		***Apply information***	
	***r***	***P***	***r***	***P***	***r***	***P***	***r***	***P***
Age (yr)	−0.4380	*P*<0.05	0.1254	*P*<0.05	0.4975	*P*<0.05	−0.0893	*P*>0.05
Sex (male)	0.0394	*P*<0.05	−0.0905	*P*<0.05	−0.1066	*P*<0.05	−0.1992	*P*<0.05
Married	−0.0045	*P*>0.05	−0.0057	*P*<0.05	0.0042	*P*>0.05	−0.0109	*P*<0.05
Education (referred to University level):								
Secondary school	−0.5015	*P*<0.05	−0.5618	*P*<0.05	−0.3809	*P*<0.05	−0.3544	*P*<0.05
High school	−0.0257	*P*<0.05	−0.0262	*P*>0.05	−0.1940	*P*<0.05	−0.4608	*P*<0.05
Ethnic groups (referred to Kazakhs group):								
Russians	0.0051	*P*>0.05	0.0017	*P*>0.05	0.0102	*P*<0.05	0.0006	*P*<0.05
Uygurs	0.0169	*P*<0.05	−0.0090	*P*<0.05	0.0088	*P*<0.05	−0.0010	*P*<0.05
Turkish	−0.0041	*P*>0.05	−0.0033	*P*<0.05	−0.0064	*P*<0.05	−0.0013	*P*<0.05
Others	0.0900	*P*>0.05	0.0760	*P*<0.05	0.0605	*P*<0.05	0.0057	*P*>0.05
Income (referred to ≥ 40,000 tenge/month):								
<15,000	−0.3065	*P*<0.05	−0.1945	*P*<0.05	−0.2112	*P*<0.05	−0.2005	*P*<0.05
15,000 – 20,000	−0.1006	*P*<0.05	−0.2091	*P*<0.05	−0.199	*P*<0.05	−0.1596	*P*<0.05
20,000 – 30,000	−0.0742	*P*>0.05	−0.1168	*P*>0.05	−0.0687	*P*>0.05	−0.0707	*P*>0.05
30,000 – 40,000	0.0056	*P*>0.05	0.0107	*P*<0.05	0.0097	*P*<0.05	0.0084	*P*<0.05
Social status (referred to low status):								
Medium social status	0.0094	*P*<0.05	0.0385	*P*<0.05	−0.0881	*P*>0.05	0.0272	*P*>0.05
High social status	0.0107	*P*<0.05	0.0472	*P*<0.05	−0.0009	*P*>0.05	0.0304	*P*<0.05
Smoking (referred to smoker):								
Non-smoker	0.3666	*P*<0.05	0.2405	*P*<0.05	0.4129	*P*<0.05	0.3807	*P*<0.05
Quit smoking	0.2704	*P*<0.05	−0.1290	*P*<0.05	0.2892	*P*<0.05	0.2965	*P*<0.05
Alcohol abuse (referred to drinking once a week)								
Never	0.3720	*P*<0.05	0.3008	*P*<0.05	0.4000	*P*<0.05	0.7701	*P*<0.05
Once a month	0.3324	*P*<0.05	0.2813	*P*<0.05	0.0516	*P*<0.05	0.0066	*P*>0.05
2 times a week	0.0917	*P*<0.05	−0.1840	*P*<0.05	0.2079	*P*<0.05	−0.0018	*P*<0.05
Almost everyday	−0.1651	*P*>0.05	−0.1337	*P*<0.05	−0.0184	*P*<0.05	−0.0049	*P*<0.05
Self-assessed health								
Excellent	0.3216	*P*<0.05	0.4991	*P*<0.05	0.3405	*P*<0.05	0.5260	*P*<0.05
Very good	0.3711	*P*<0.05	0.4513	*P*<0.05	0.2553	*P*<0.05	0.5370	*P*<0.05
Good	0.3572	*P*<0.05	0.4848	*P*<0.05	0.3264	*P*<0.05	0.4917	*P*<0.05
Bad	−0.3690	*P*<0.05	0.4103	*P*<0.05	0.2842	*P*<0.05	−0.4601	*P*<0.05
Very bad	−0.3572	*P*<0.05	0.3008	*P*<0.05	0.2987	*P*<0.05	−0.3722	*P*<0.05

The younger group indicated the access to health information as easy, and the appraisal of health information as difficult or very difficult. At the same time, for the older respondents, it was more difficult to access health information and easier to appraise it.

There was also some correlation between age groups and health literacy, especially in the domains of accessing and appraising information. Data presented in the Table 3 indicate also that non-smoker respondents, people who quit smoking and never drink alcohol have better scores in access, understanding, and appraising and applying health information than smokers or respondents who drink alcohol.

Interesting results are seen when consider the association between the self-assessed health status and health literacy. Higher literacy rate, especially in understanding and applying health information, higher rate of self-assessment of the health (Table 3).

## Discussion

Kazakhstan is a newly independent state with a more than 17.5 million inhabitants. A middle-income country with the prevalence of rural population, literacy rate of 99.8% (2015). In 2015, life expectancy at birth made 70.2, infant mortality rate – 20.3 per 1000 live births (33).

The stratified sampling method led to the accordance of the sample distribution to the Kazakhstani rural population distribution in terms of sex, education and income (34).

The measuring health literacy of target populations is essential tool for planning health promotion activities. The purpose of this study was to assess the health literacy of the rural population for the development of targeted health education programs. The use of the HLS-EU model allowed us to assess functional, communicative and critical levels of the health literacy.

The study findings suggest that the rural population in Almaty region of Kazakhstan has different health literacy scores in various competences -accessing, understanding, appraising and applying health information. This is in accordance with the results from other studies (6–8, 16, 35, 36). In general, the studied population has more difficulties in the competences than European Union countries (15, 16).

Between these competences, the respondents have bigger difficulties in accessing and appraising health information than in understanding and applying them. These difficulties in the competences are different scale depending on the health domain: the competences in disease prevention are perceived not such difficult as in health care; or appraising information in disease prevention is more difficult that in health promotion. At the same time, understanding information in disease prevention was the easiest for the respondents, however applying information in disease prevention was the most difficult one.

The present research shows that there is some social gradient in health literacy. In terms of demographic, social and economic determinants of health literacy competencies, the study found some determination. The health literacy competences were heavily dependent on age of the respondent. For young people accessing information was much easier than understanding and appraising health information. At the same time, for older part of the population appraising and applying health information was easier than accessing information. The development of mobile internet and higher ability of younger respondents to use it and more careful attitude to health and more ordered life of older people play a certain role (10, 26) and these findings are in accordance with other study results (13–16).

The research results did not find certain association between sex and health literacy as shown in much other literature (6, 7, 16).

The research findings indicate the certain positive association between education level and HL indexes, especially in accessing and understanding information the domains of health care and disease prevention, related to functional health literacy (5, 18). On appraising and applying information related to critical health literacy (5, 18), the respondents with higher education level have almost the similar scores as the respondents with lower education level.

The study results show that lower social and economic status leads to lower health literacy that is consistent with findings of other studies (6–8, 16, 35). Some variations of health literacy in income group are found in all four competencies, particularly in accessing and understanding health information. However, these variations are not such big as for the education level.

The present study results suggest that the education level among respondents of different ethnic origins differs (36). The highest share of people with University degree met in the Russian respondents (13.9%), followed by the Uighurs and Kazakhs (9.1%). Uighur respondents had the highest share of people with vocational education (44.5%), and Russian-slightly lower (43.5%). There is the highest percentage of persons with incomplete high school was seen in Uighur ethnic group. The smallest percentage of persons with incomplete secondary education met the Turkish ethnic group.

Despite the fact of these differences, there is no association found between health literacy and ethnic origin of respondents.

The analysis of effects of current health behavior on health information competences respondents suggests strong negative correlation between health literacy and smoking and alcohol abuse, especially in appraising and applying health information.

According to research data, respondents’ self-assessment of own health depends on their understanding and applying health information: most of respondents, who have better understanding and applying health information, assess own health as “excellent” or “good”. Their good health could be a result of their health literacy. Health literacy motivates people to take healthy decisions in their everyday life.

Health information has effect across the rural population in Kazakhstan and improving health literacy will positively influence on their health.

The HLS-EU questionnaire is an effective instrument of the health literacy measurement and can be used for these purposes among Kazakhstani population since it provides an in-depth insight into health literacy as a multidimensional concept. In addition, Kazakhstan has specific historical background connected to soviet period and has literacy level similar to European.

Shown above limited health literacy and social gradient in health literacy in rural population should represent important challenges for health policy and practice in Kazakhstan.

Since the survey was self-administered, adults with inadequate reading abilities may not be included. In addition, it is likely that adults from ethnic minorities perceive more difficulties with health information, and hence the results might underestimate the health literacy skills of the adult population.

## Conclusion

The rural population of Almaty region in Kazakhstan has overall low health literacy (at inadequate and problematic levels). It demands more attention from the local and central government and policy makers and requires targeted health education interventions. Different socioeconomic groups of this population have different health information competences in healthcare, disease prevention and health promotion domains. Low educated people and with lower income have lower health literacy in comparison to respondents with higher education level and higher income and these results are in accordance with other studies. Respondents with higher health literacy have higher rate of self-assessed health. The rural population would benefit from improving the accessibility and enhancing the content of the health information, especially in the health promotion and healthcare domains.

## Ethical considerations

Ethical issues (Including plagiarism, informed consent, misconduct, data fabrication and/or falsification, double publication and/or submission, redundancy, etc.) have been completely observed by the authors.
